# ‘Green podiatry’ - reducing our carbon footprints. Lessons from a sustainability panel

**DOI:** 10.1186/s13047-021-00497-1

**Published:** 2021-11-29

**Authors:** Angela Margaret Evans

**Affiliations:** Discipline of Podiatry, School of Allied Health, Human Services and Sport, La Trobe University, Melbourne, Victoria 3086 Australia

**Keywords:** Climate change, Footprint, Healthcare, Carbon, Podiatry, Emissions, Health, Green

## Abstract

**Background:**

The eyes of the world will be on COP26 as it meets in Glasgow in November, 2021. Our planet is displaying weather extremes due to climate change which cannot be ignored, and which are deleterious for people’s health. Ironically, healthcare contributes to climate change, contributing approximately 5% of carbon emissions globally. Climate change due to global warming is ‘*the biggest global health threat of the 21st century’*.

**Main body:**

The Australian Podiatry Association conference held a sustainability panel, hearing perspectives of industry and science, medicine and sport, fashion, and retail. Content unified a broad planet and human health message, which is highly relevant for podiatrists. Key themes included waste as a resource, exercise as evidence-based intervention, responsibility and circular economy recycling principles for end-of-life product (footwear) purchases, and wider ethical considerations of footwear and clothing.

The Anthropocene origin of climate change requires humanity to *collaborate and* to live more sustainably. *Innovation* is essential for better energy modes, cleaner air, human health and earth care.

Green Podiatry joins the concerted activity of medical and health groups within Australia. The UK’s NHS is an exemplar in this area, having already reduced healthcare emissions by 35%, and aiming for net zero by 2045, and perhaps sooner.

**Conclusion:**

People are increasingly concerned about climate change, and COP26 is an important and imminent meeting for human and planet health.

This commentary on **Green Podiatry** directs us all to lighten our carbon footprint. A final, and forthcoming commentary will outline practical ways of positively incorporating climate change communication into the clinical setting.

**Supplementary Information:**

The online version contains supplementary material available at 10.1186/s13047-021-00497-1.

## Background

Health care’s (HC) carbon footprint is equivalent to 5% of global net emissions, and contributes to climate change [1].

**Table 1:**
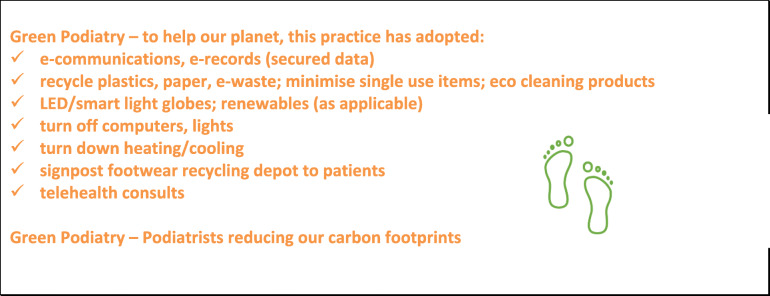
A suggested check-list for display in Green podiatry practices, and tips for home. Selecting whichever actions apply, simple *Green Podiatry* signage can start conversations about health and climate change with patients, and designate more sustainable clinics for consumers.

As COP26^1^ approaches, emissions reduction targets remain well behind in Australia, where HC emissions exceed 7%. Hospitals contribute almost half [2], with energy use 60–70% of all HC emissions, mainly from fossil fuels.

‘Health Care without Harm’ is a principle that all health practitioners would, it is hoped, aspire to [3]. Hence, we must act to reduce health-harming climate change, from increased greenhouse gases, and the resulting effects, as recently (June – August 2021) seen in Germany (floods), Siberia (fires), polarised temperatures of 55C in Canada, and 18C in Antarctica [4, 5].

COP26 may be the last chance to agree on measures that could limit global warming to 1·5 °C. There is unique opportunity to align the global recovery from COVID-19, with the response to climate change to improve public health, create sustainable economies, and protect the planet [6].

## The sustainability panel

The finale to the Australian Podiatry Association 2021 conference, attended online by over 800 delegates, saving tonnes of CO_2_ emissions, was a sustainability panel. This forum addressed sustainability from the perspectives of industry and science, medicine and sport, fashion, and retail, and united a ‘no waste’, and broad planet and human health message, with a complementary podcast [Additional file 1 - Link 1]. It was fascinating, and highly relevant for podiatrists.^2^

Scientia Professor Veena Sahajwalla [Director of the Centre for Sustainable Materials Research and Technology at University of New South Wales; Australian Research Council Laureate Professor]

- Waste is a resource: micro-resources and materials to be recycled, re-used, reformed at end of use to be fit for a new purpose as functional products, eg ‘green steel’ from old car tyres.
- Averting products from landfill, and manufacturing quality products from waste (within Australia) can realise environmental, social, and economic benefits simultaneously.
- A pilot collaboration into ‘green foot orthoses’ has begun.

Dr. John Orchard AM [Sport & Exercise Physician; Adjunct Professor, Sydney School of Public Health]

- Exercise is demonstrated as effective treatment for most of Australia’s National Health Priority Areas, viz. cardiovascular disease, cancer, arthritis, back pain, depression, osteoporosis, and diabetes [Additional file 1 - Link 2]
- Podiatrists need to consider exercise as first-line treatment, which is evidence based and valuable for every patient, ahead of foot orthoses [7]
- Is treatment even necessary [8]? If not necessary, or of low value, not evidence-based, and especially if also carbon emission ‘heavy’, it is wise to eliminate wasteful interventions, as these do not help patients, and harm the environment, eg
  - customised foot orthoses for non-painful paediatric flatfeet [9–11], plantar heel pain [12]
  - imaging for calcaneal apophysitis [13, 14]
- Health professionals need to drop non-evidence-based interventions, and c*hoose wisely* [Additional file 1 - Link 3].

Shaun Bajada [Executive Director, Australian Sporting Goods Association (ASGA)]

- Australians purchase more than 25 million pairs of sports shoes annually.
- ASGA’s shoe recycle programme is called ‘save our soles’ (SOS). SOS has recycled more than 115,000 shoes, converting these to gym mats, flooring, playground turf.
- SOS operates as a circular economy, taking responsibility for end of life of purchased products. The principle is to be carbon neutral.^3^
- SOS notes that increasingly, sustainability is the cornerstone of the consumer’s mind.

Nick Savaidis [Founder and Director, Etiko]

- The fashion industry is the second largest polluting industry after the oil industry [15].
- Etiko’s long term goal is to keep its carbon footprint minimal
- Adopted a circular economy model, with a ‘take back’ program for old footwear
- Etiko address their total impact, considering workers, farmers in the supply chain – promoting ethical production, as well as environmental sustainability, as a Fair Trade Certified brand [Additional file 1 - Link 4].

## Green podiatry can improve health

The 2019 Australian Podiatry conference theme was ‘Physical activity, Feet, and wider health’. In 2021, the virtual sequel theme, was ‘Innovation and Collaboration’. Given the Anthropocene origin of climate change [5, 16], we need to *collaborate* to live more sustainably. *Innovation* is a big part of this, and drives developments in better energy use and earth care [17].

Podiatrists can make a ***Green LEAP*,** and be actively involved in sustainable HC (Table 1):

- **L**ead on environmental sustainability
- **E**ducate themselves about environmental sustainability
- **A**dvocate for better healthcare sustainability
- **P**articipate in environmentally sustainable workplaces [17].

## In the global context, it is ‘all aboard’

The 6th Assessment Report of the Intergovernmental Panel on Climate Change, 2021 has declared a *Code Red* for humanity [Additional file 1 - Link 5]. The Paris agreement, the United Nations, and the WHO all acknowledge the environmental impacts from HC from:

- consumption of energy and resources
- greenhouse gas emissions
- use/disposal of toxic chemicals
- production of waste/wastewater.

A time of global reckoning approaches with COP26 [Additional file 1 - Link 6].

## ‘Down under’ – and all over

Doctors for the Environment Australia (DEA) recognise that human health and wellbeing require an environment that is pollution-free, able to provide nutritious food, rich in biodiversity, and able to sustain current and future generations. DEA have adopted the mantra: *‘healthy planet, healthy people’* [Additional file 1 - Link 7].

The Climate and Health Alliance (CAHA) is a coalition of HC stakeholders, from medicine, nursing, public health, social work, and psychology, as well as HC service providers, research and academic institutions, and health consumers. CAHA have a guide for health professionals to act on climate change [Additional file 1 - Link 8]. The report, entitled *Real, Urgent and Now,* found that 86% of health professionals want immediate action on climate change [Additional file 1 - Link 9].

The Australian Podiatry Association has commenced a ‘green’ strategy [Additional file 1 - Link 10]. By applying the principle of responsible consumption and production, podiatrists can reduce emissions at work, and at home (Table 1). The promotion of healthy feet for walking, running, and cycling, enables carbon-neutral transport, and also benefits health.

The UK’s NHS has led the world in developing a strategy for sustainability, with a Sustainable Development Unit (SDU) and carbon reduction targets [Additional file 1 - Link 11]. The NHS is an exemplar, having cut its carbon footprint by 25% since 1990, and by 35% since 2008 [18–21], mostly by less reliance on fossil fuels, and decarbonising UK electricity. Air quality has improved, financial savings made, and 92% of NHS staff are supportive.^4^ Clinical care is the next NHS challenge, as further carbon footprint reductions require clinical care to change. In some areas, opportunities for reuse are limited (eg infection control standards). This requires innovation, as do single use items [22], and avoiding unnecessary or low value care [Additional file 1 - Link 12]. The Royal College of Physicians have produced a guide for health professionals to reduce waste and the NHS target is to reduce carbon emissions by 80%, by 2045.

People are increasingly concerned about climate change, and more consumers prefer sustainable practices [6, 23]. Podiatrists are in a great position to act and to educate [24]**.** Leadership needs passionate people to role model ‘green’ behaviours, and HC students are a vital inclusion, with many already concerned about their futures [25, 26].

## The environment cannot wait

Eco-ethical leaders are needed in academia and HC to join environmental advocates and activists [24]. Using the WHO physical activity guidelines [7], underpins the importance of exercise for health, and to teach patients about exercise benefits for their health, as well as the planet.

## Conclusions

**“Climate change is the biggest global health threat of the 21st century”** [27**]**, yet with vision, has the potential to be, ‘the greatest healthcare opportunity of the 21^st^ century’ [Additional file 1 - Link 13].

COP26 is pivotal for global warming to be reined in. **Green Podiatry**, as part of green HC, directs us all to lighten our carbon footprint. The next commentary will focus on talking with patients about green HC, and the need to eliminate low value care, to promote exercise, sustainable footwear, and telehealth. This is important, as people are worried [6, 28].

## Supplementary Information

**Additional file 1**

## Data Availability

N/A

## Abbreviations

CO_2_: carbon dioxide
WHO: World Health Organisation
UN: United Nations
DEA: Doctors for the Environment Australia
CAHA: Climate and Health Alliance
HC: Healthcare
COP26: Conference of Parties, Glasgow, November 2021
NHS: National Health Service

## Footnotes

1 Conference of the Parties - meeting of the 197 members to the United Nations Framework Convention on Climate Change

2 Sustainability panel recording: https://www.smart.unsw.edu.au/news-events/news/podiatry-and-sustainability

3 SOS collection depots: https://www.asgasosinitiative.com.au/collection-locations/

4 Nick Watts, 14/9/2021, https://dea.org.au/event/australian-medical-association-doctors-for-the-environment-australia-webinar/
