# Supplementary material for: ‘Green podiatry’ - reducing our carbon footprints. Lessons from a sustainability panel
Source: J Foot Ankle Res. 2021 Nov 29;14:59. doi: 10.1186/s13047-021-00497-1 (PMC8628046; doi:10.1186/s13047-021-00497-1)
Supplement: Supplementary file 1 — Additional file 1 [file 13047_2021_497_MOESM1_ESM.docx]

**Additional file -** Links

1. Sustainability podcast: <http://www.buzzsprout.com/956374%C2%A0>

# Orchard J, Drop wasteful procedures to do our bit for climate, Medical Republic, March, 2021: <https://medicalrepublic.com.au/drop-wasteful-procedures-to-do-our-bit-for-climate/42620>

# Choosing wisely: <https://www.wiserhealthcare.org.au/wiser-carbon-neutral/>

# Etiko: <https://etiko.com.au/pages/our-story>

5. IPCC, 2021: Climate Change 2021: The Physical Science Basis. Contribution of Working Group I to the Sixth Assessment Report of the Intergovernmental Panel on Climate Change [Masson-Delmotte, V., P. Zhai, A. Pirani, S.L. Connors, C. Péan, S. Berger, N. Caud, Y. Chen, L. Goldfarb, M.I. Gomis, M. Huang, K. Leitzell, E. Lonnoy, J.B.R. Matthews, T.K. Maycock, T. Waterfield, O. Yelekçi, R. Yu, and B. Zhou (eds.)]. Cambridge University Press: <https://www.ipcc.ch/report/ar6/wg1/>

6. COP26: <https://ukcop26.org/>

7. DEA: <https://www.dea.org.au/>

8. CAHA guide: [www.caha.org.au/act](http://www.caha.org.au/act)

9. CAHA RUN report, 2021: <https://www.caha.org.au/run>

10. Australian Podiatry Association: <https://stride.podiatry.org.au/wp-content/uploads/2021/09/202109-apoda-stride-issue.pdf> (page 12)

11. NHS: <https://www.kingsfund.org.uk/projects/time-think-differently/trends-sustainable-services>

12. Wiser healthcare: <https://www.wiserhealthcare.org.au/wiser-carbon-neutral/>

13. Abbott J; Could addressing climate change be the greatest opportunity for the 21st century? *University of Cambridge* [<https://studentsforglobalhealth.org/2020/06/23/could-addressing-climate-change-be-the-greatest-opportunity-for-the-21st-century/>].

**Further Resources**

- Green tips for reducing emissions at work, and at home: <https://www.un.org/sustainabledevelopment/sustainable-consumption-production/>
- Telehealth tips - Evans AM. Sustainable healthcare – Time for ‘Green Podiatry.’ *J Foot Ankle Res* 2021; 14: 45.
- Knibbs LD, Woldeyohannes S, Marks GB, Cowie CT. Damp housing, gas stoves, and the burden of childhood asthma in Australia. *Med J Aust* 2018; 208: 299–302.
